# Insects as Valuable Sources of Protein and Peptides: Production, Functional Properties, and Challenges

**DOI:** 10.3390/foods12234243

**Published:** 2023-11-24

**Authors:** Fatin Fayuni Binti Hasnan, Yiming Feng, Taozhu Sun, Katheryn Parraga, Michael Schwarz, Mohammad Zarei

**Affiliations:** 1 Department of Food Science and Technology, School of Industrial Technology, Faculty of Applied Sciences, Universiti Teknologi MARA, Shah Alam 40450, Malaysia; fatinfayuni97@gmail.com; 2 Virginia Seafood Agricultural Research and Extension Center, Virginia Polytechnic Institute and State University, Hampton, VA 23669, USA; yimingfeng@vt.edu (Y.F.); taozhu@vt.edu (T.S.); kparraga@vt.edu (K.P.); mschwarz@vt.edu (M.S.)

**Keywords:** insects, protein, protein hydrolysate, bioactive peptides, entomophagy, nutritional value, functional properties, sustainability, alternative protein sources, food production methods

## Abstract

As the global population approaches 10 billion by 2050, the critical need to ensure food security becomes increasingly pronounced. In response to the urgent problems posed by global population growth, our study adds to the growing body of knowledge in the field of alternative proteins, entomophagy, insect-based bioactive proteolysates, and peptides. It also provides novel insights with essential outcomes for guaranteeing a safe and sustainable food supply in the face of rising global population demands. These results offer insightful information to researchers and policymakers tackling the intricate relationship between population expansion and food supplies. Unfortunately, conventional agricultural practices are proving insufficient in meeting these demands. Pursuing alternative proteins and eco-friendly food production methods has gained urgency, embracing plant-based proteins, cultivated meat, fermentation, and precision agriculture. In this context, insect farming emerges as a promising strategy to upcycle agri-food waste into nutritious protein and fat, meeting diverse nutritional needs sustainably. A thorough analysis was conducted to evaluate the viability of insect farming, investigate insect nutrition, and review the techniques and functional properties of protein isolation. A review of peptide generation from insects was conducted, covering issues related to hydrolysate production, protein extraction, and peptide identification. The study addresses the nutritional value and global entomophagy habits to elucidate the potential of insects as sources of peptides and protein. This inquiry covers protein and hydrolysate production, highlighting techniques and bioactive peptides. Functional properties of insect proteins’ solubility, emulsification, foaming, gelation, water-holding, and oil absorption are investigated. Furthermore, sensory aspects of insect-fortified foods as well as challenges, including Halal and Kosher considerations, are explored across applications. Our review underscores insects’ promise as sustainable protein and peptide contributors, offering recommendations for further research to unlock their full potential.

## 1. Edible Insects

Insect-based food sources are frequently consumed in tropical and subtropical regions, whereas their acceptance is notably limited in Western countries [1]. Insects have a high amount of protein, fat, and minerals, positioning them as a promising food source and ingredient option for enhancing the quality of various food products, including bread, pasta, protein bars, snacks, and processed meat products. In addition, insects have significant potential as a sustainable solution for future nutrition demands because they are renewable natural resources available all year round and have low environmental impacts with enhanced food safety [2,3].

In contrast to conventional primary protein sources utilized for human food and animal feed, insects exhibit high protein content and well-balanced amino acids. Notably, insect farming demands reduced land, water, and gas emissions compared to traditional protein sources (Table 1) [4,5].

The main obstacles to the expanding insect production industries are inadequate consumer acceptance and the need for well-defined regulations governing insect-based food and feed. Such impediments could hinder the widespread production and promotion of insects as a viable food source, as specific stakeholders perceive the associated processes to be both costly and time-consuming [9]. According to Johnson [10], insect-eating seems to be culturally universal; the only factors that change are the location, the population of insects, and ethnicity. Notably, ancestral human populations in Africa likely incorporated insects into their diets, a tradition observed in present-day primates that exhibit an enthusiastic inclination towards insect consumption. These food sources are likely served as routine, ritual, or emergency. Forest insects are commonly gathered from various regions to be part of human diets. Unfortunately, in conjunction with the exploitation of forest insects, the oversight of forest plant management has been minimal in most cases. Furthermore, it is worth noting that the domestication of insects has been limited primarily to a select few species, such as silkworms and bees. Larvae and pupae are the most often consumed insects, typically undergoing minimal processing prior to consumption. Table 2 Provides a comprehensive compilation of edible insects, presenting these species organized by taxonomic orders, common English names, and Vernacular names.

Almost 2000 species of insects are documented worldwide to be utilized as food for humans, and the number is constantly increasing. Approximately 49% of these edible insect species are beetles and caterpillars; 14% are bees, wasps, and ants; and 13% are grasshoppers, locusts, and crickets; Hemipterans (10%); dragonflies (3%); termites (3%); and flies (2%) [11,13]. Four insect orders prevail in the Coleoptera, Hymenoptera, Orthoptera, and Lepidoptera sequence of classes, representing 80% of the species consumed (Table 2) [10,14].

Geographically, the utilization of insect species as food is most extensively documented in the regions of America and Africa, with 679 and 524 species, respectively. Asian countries and Pacific nations have also documented the consumption of over 500 insect species. Notably, the actual count of consumed species in Asia is likely to exceed this recorded statistic, as there appears to be less rigorous study in Asia and the Pacific nations as compared to studies in Africa and the Americas [10].

### Entomophagy around the World

**Figure 1:**
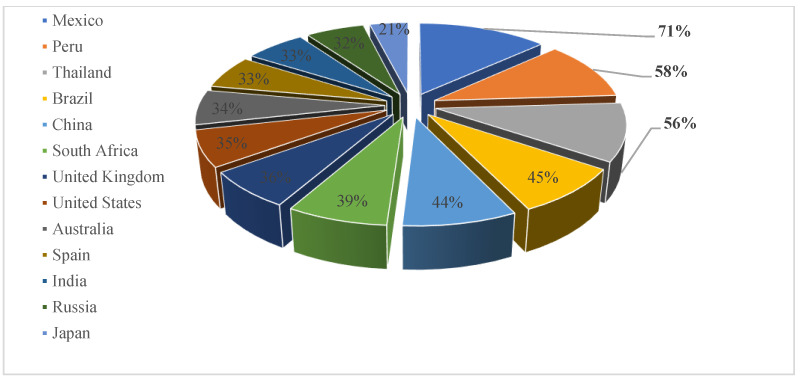
Global willingness for insects’ consumption.

Insects have been utilized as a source of sustenance across diverse regions worldwide. Archeological evidence shows that entomophagy, the practice of consuming insects, dates to humanity’s earliest days and has persisted as a crucial food source. Although there is no dedicated study providing the precise percentage of consumers’ willingness to eat insects across various global regions, existing research, including a survey with 8190 respondents from 13 countries conducted by Castro and Chambers IV [15], showed that Mexico, Peru, and Thailand revealed the highest willingness to eat insects with 71%, 58%, and 56% of respondents, respectively. However, Japan, Russia, and India showed the lowest willingness to consume insects, with 21%, 33%, and 33%, respectively (Figure 1).

Nowadays, in Africa, numerous grasshoppers, termites, and large moth caterpillars are commonly consumed, including Lepidoptera, mopane worm/caterpillar *(G. belina*). Other studies also reported that insects are also consumed by people in South and Central America, Australia, the Middle East, and Asia [16]. For instance, in the Philippines, the most common insect served in restaurants is mole crickets, and to catch them, farmers flood their lands. On the other hand, the Thais consume crickets, grasshoppers, bugs of water, larvae, and dragonflies. Six insect species have even been featured in a publicly available handbook on nutrient foods by the Royal Thai Government. In addition, fried insects are being sold at Bangkok’s roadside food booths. The Chinese also consume various insects, many of which are used for therapeutic applications, such as silkworms, crickets, and ants, which are believed to have medicinal properties and health benefits. Similar consumption trends may be seen in Japan, the Republic of Korea, and Indonesia [17,18,19,20].

The sago grub has been one of the most prominently consumed insects in Sabah, Malaysia. Derived from the larvae stage of the palm weevil (Coleoptera), the grub is named butode, wutod, or tobindok among the Kadazandusun in western Sabah. At the same time, it is also called sungut in Telupid. After falling and decomposing for approximately two or three months, the creamy yellow larvae are gathered from the sacral sago instead of the sacral trunk. In areas with limited sago palms, the falling trunks of cocoa palms may be used [10].

Furthermore, planters and farmers working in rice fields commonly find different types of grasshoppers as food (Orthoptera). Among these types, the most widely observed ones include pointed-nose grasshoppers, short-horned grasshoppers, leaf-like grasshoppers, and valanga grasshoppers. These grasshoppers are typically collected after the removal of paddy crops. Unlike sago grubs, which are commercialized, grasshoppers are primarily harvested for household consumption and are not traded in the market. Their preparation is uncomplicated. First, they are lightly seasoned, followed by a simmering process until the moisture evaporates. In certain instances, larger grasshoppers are deep fried to achieve a smooth texture, similar to fried creams. They are usually served as one dish and not combined with meat or vegetables.

The mole cricket, *Gryllotalpa longipennis* (Orthoptera), is also found in the paddy field. In Malaysia, it is known as “suruk” or “tongook”, which translates to “hide”. This insect is suitable for subterranean living. Similar to the mole, the forelegs dig fast into the soil, and they frequently feed on paddy roots. Thus, mole crickets are typically gathered before plowing when the land is planted. While this mole cricket can be prepared using various cooking techniques, it is frequently pan-fried without the need for added oil. This culinary practice could stem from both its delectable taste and the perception that it offers a healthier dietary choice [17].

The acceptance of insects as a consumable food source is influenced by many factors, including nutrient content, especially the protein, taste, flavor, appearance, and palatability of insects. Additionally, external factors such as the insects’ availability, convenient pricing, and conducive social environment play a pivotal role in shaping consumer attitudes toward insect consumption, as reviewed by Meyer-Rochow, et al. [21]. The grasshopper (*Ruspolia differens*), as a typical and traditional delicacy, is a valuable source of nutrients in Kenya, Tanzania, and Uganda. In these regions, consumers prefer salted, boiled, smoked, or deep-fried grasshoppers in cottonseed oil compared to the other types [21,22]. Termite soldiers with long bodies were in great demand and highly preferred over alate forms in Kenya. Consumers evaluate the insect stock for freshness, the presence of legs, cleanliness, species type, and oil content at the local market before purchasing termites [23].

## 2. Insects Nutritional Value of Edible Insects

### 2.1. Protein Content of Edible Insects

The nutritional value of insects varies significantly across species, developmental stage, diet, growth environment, sex, and measurement methods [21]. However, researchers generally agree that insects are extremely rich in protein. On average, the protein content of edible insects ranges from 35–60% dry weight or 10–25% fresh weight [24,25,26], and the content found in certain insects is higher than some plant-based proteins. Therefore, insect-derived protein presents itself as a viable and premium protein source, particularly beneficial for individuals struggling with inadequate nutritional intake due to protein deficiencies. Indeed, nutritionists are the dominant research group on food insects, driven by a desire to solve protein-deficient dietary concerns [14]. Table 3 shows an overview of the protein content in select edible insects on a dry-weight basis. As shown, various insects show different protein contents based on their species, development stage, etc. Therefore, the protein content of an insect, depending on the species, is in a range rather than an absolute amount.

One crucial factor needs to be considered while analyzing the insect’s protein content. Considering the possible overestimation linked to the commonly used 6.25 nitrogen-to-protein conversion factor is essential when assessing insect protein content. This component, which was first found to be appropriate for meat, might not apply to insects because of the substantial amount of fibrous chitin that is present in their cuticles. Recent research has shown that the chitinous composition of insect cuticles might cause an overestimation of protein content when applying the traditional conversion factor [35,36,37]. We highlight the need to investigate other aspects in light of this, as indicated by research on particular insect species (e.g., field cricket and house cricket, which have recommended values of 5.00 and 5.09, respectively) [35] or the study performed by Janssen, Vincken, van den Broek, Fogliano, and Lakemond [36], which suggested a conversion factor of 5.60 ± 0.39 after protein extraction and purification for the larvae of three insect species studied. This approach reduces the possibility of overestimation by ensuring a more accurate representation of insect protein content and recognizing the importance of species-specific characteristics.

In a review paper, Hawkey, et al. [38] compared the nutrient value of ten different insects with soybean meal. Their study showed that the relative protein level was varied, with the highest amount of protein content in the adult locust with 76% (Dry matter-based) crude protein, and the crude protein of the reviewed insects ranging from 23% to 76%. Soybean meals with 55.2% protein showed a lower crude protein than most insects, indicating the importance of insects as a valuable source of high-quality proteins. However, the abundant protein content observed could potentially be influenced by the presence of the nitrogen-rich polysaccharide chitin, which was also measured as protein. Because of that, Janssen, et al. [36] proposed using protein factor 5.60 instead of 6.25 for determining the crude protein content in insects to avoid overestimating the protein content.

In a separate comprehensive analysis, Rumpold and Schlüter [33] conducted an assessment of protein content across 236 edible species from various insect orders. According to their review paper, the protein content ranged from 6% to 77% of dry matter, considered significant across insect groups and between them. Moreover, considering the average contents of different orders of insects, the main components of insects were protein and fat, followed by fiber, nitrogen-free extract (NFE), and ash, in no particular order. Furthermore, based on the comparison conducted by Van Huis, et al. [39] and the literature reviewed by Churchward-Venne, et al. [40], the protein content of select insect orders is comparable to the protein content of cattle, fish, egg, soy, and milk. Remarkably, the protein content of grasshopper (*Sphenarium purpurascens*) (35–48%) is higher than conventional protein sources such as lamb (16.32–17.91%), beef (17.17–32.27%), pork (17.43–21.10%), chicken (16.52–22.50%), turkey (18.73–21.84%), and fish (16.38–29.40%) [41,42].

### 2.2. Amino Acid Composition

Insects often compare well with conventional sources of protein, for example, soy and fish. A large percentage of the protein within the exoskeleton is chemically tied and thus may not be accessible. Furthermore, exoskeletons include polysaccharide chitin containing nitrogen, which can potentially lead to an overestimation of the overall protein composition of insects when evaluated based on nitrogen content. To address this, a nitrogen-to-protein conversion factor of 5.60 for a range of insect species may be more suitable than the standard ratio of 6.25. Alternately, by removing the nitrogen contained in the chitin exoskeleton, the protein composition of insects may be reassessed. Insects are usually considered an essential source of the essential amino acid (EAA). The amino acid content of selected insect species (Milligrams per gram of protein), fish, and beef protein content are presented in Table 4. Even though species-developed levels vary with EAA at particular levels, they generally compare well with conventional protein sources used in animal feed and high-quality sources of human protein such as meat, milk, and fish [38].

A total of 77% to 98% for 78 different species was indicated to span through various orders. However, the assessment of protein digestibility remains incomplete across all insect species. The apparent protein digestibility for mealworm and black soldier fly was reported as 60% and 51%, respectively. Intriguingly, the crude proteins of the mealworm and black soldier fly digestibility appear to exhibit an inverse relationship with their chitin concentration, as elucidated through in-vitro digestive methods. Pretorius supplied corn-based foods that were additional to fly larval or pupae meal by 50%. They showed greater total pulp digestibility with the pupae meal for crude proteins and specific amino acids [38].

In another review, Tang et al. [52] stated that insects are essential animal protein suppliers at all stages of life. Table 4 depicts the amino acid spectrum of edible insects. The composition of crude protein is commonly 40% to 75% by dry weight basis, with typical values per order of 33% to 60%. Edible insects typically have higher levels of crude protein compared to conventional protein, although their compositions of amino acids are often similar. Insects’ meals are an important source of amino acids with high digestibility of 76% to 96%. The essential and non-essential amino acid composition of a widely consumed species is demonstrated by the World Health Organization for adults.

Some insects exhibit deficiencies or extremely low levels of methionine, cysteine, and tryptophan. If such insects are consumed as the main part of the diet, the food or feed should be well balanced with other ingredients. Blattodea tend to possess higher concentrations of lysine, valine, methionine, arginine, and tyrosine in comparison to other insect varieties. The quantity of leucine in Coleoptera is larger than in other types of protein sources, including beef meat. Similarly, the quantity of phenylalanine in Hemiptera is typically larger than in other conventional protein sources. Among the various developmental stages of edible insects, nymphs emerge as a particularly abundant source of virtually all essential amino acids. Arginine, a valuable amino acid recognized for its cardiovascular benefits, as well as its immune system-boosting properties, is found abundantly across these insect species.

### 2.3. Fatty Acids Composition

As Table 5 illustrates, the quantity of insect-related fat within and across species may vary considerably. Typically, larval invertebrates exhibit significantly higher fat levels than adults on a dry-matter basis. Triacylglycerol and phospholipids in various forms represent 80% and 20% of the total fat content, respectively. Compared to fish or soy meal, to utilize insects in animal feeds, a part of the fat content will have to be removed. This extracted fat can be utilized as an additional component. Table 5 summarizes the reported fatty acids compositions of several insect species.

Generally, insects have a spectrum equal to other animal species in saturated and monounsaturated fatty acids and polyunsaturated fatty acids (PUFA). Among saturated fatty acids, palmitic acid (C16:0) is the primary constituent, often accompanied by varying proportions of stearic acid (C18:0). The principal monounsaturated fatty acid is oleic acid (C18:1); however, certain species can have a considerable accumulation of palmitoleic acid (C16:1) in rare cases. In general, insects with lower levels of n-3 alpha-linolenic acid are relatively high in n-6 PUFA (n-6 C18:2) and linoleic acid (C18:3). However, few indications show that insects can manufacture long-chain N-3-PUFAs, such as Eicosapentaenoic, EPA (C20:5), and DHA (C22:6), which are oily fish-related acids. Alterations in food can change the total fat and fatty acid content of different insect species. For instance, introducing rich PUFA sources into the diet of mealworms has been demonstrated to yield a significant increase in their PUFA content, as observed in the study by Hawkey, Lopez-Viso, Brameld, Parr, and Salter [38].

Meanwhile, a study by Tang, et al. [52] stated that insects are commonly rich in fats. Table 5 presents the fatty acid spectrums of typical edible insects based on dry matter. Other fatty acids, such as odd-numbered fatty acids, even-numbered saturated fatty acids, and certain unsaturated fatty acids, are not listed in Table 5 but have been identified in trace amounts of other acids. Several acids are deemed insignificant. In the immature stage, the fat contents of insects vary from 8% to 70% based on the dry weight. However, across diverse meat sources encompassing various insect species, a remarkable similarity is observed in their fatty acid compositions. Furthermore, it is observed that the fat content in Lepidoptera and Heteroptera is greater than that of other edible insects.

Insect fat primarily consists of triacylglycerol, with saturated fatty acids (SFAs) and monounsaturated fatty acids (MUFAs) accounting for over 80% of their composition. Among SFAs, palmitic acid and stearic acid are the predominant components in various stages of insect development. SFAs usually are greater in their content than MUFA among adults. The most frequent insect MUFA in the human diet is oleic acid, a monounsaturated fatty acid. It contributes to human blood pressure reduction and has a solid potential to cure inflammatory, immunological, and cardiovascular disorders. Although there is a risk of overtaking the SFAs in diet, maturing insects provide a better supply of polyunsaturated fatty acids (PUFAs) than traditional sources like pork and beef. Compared with other insect orders, Orthoptera is the best linoleic acid source [53,54].

**Table 5:** Fat and fatty acids composition of some insects based on dry weight (g/100 g dry-weight basis).

Common Name	Fat Content	Saturate d Fatty Acids (SFA)	Monounsaturated Fatty Acids (MUFA)	Polyunsaturated Fatty Acids (PUFA)	Linoleic Acid (18:2)	Alfa Linolenic Acid (18:3)	Arachidonic Acid (18:4)	Reference
Winged termite	44.82	35.05	52.77	12.18	10.75	1.43		[55,56]
White spotted flower beetle	26.70	23.61	95.20	10.40	9.10	0.40	0.70	[27,57]
Desert locust	13.00	25.30	39.35	26.28	14.04	11.35		[58]
Dung beetle *	13.50	733.46	85.65	1514.32	-	39.82	934.95	[59,60]
Black soldier fly larvae	35.00	36.20	28.70	35.00	13.00	1.70	-	[61,62,63]
Sugarcane termite	46.00	32.17	56.10	11.73	11.54	0.20	-	[56]
Tropical house cricket	20.00	33.74	34.33	31.91	29.78	2.13	-	[58]
Guizhou black ant	15.20	23.90	72.40	3.70	2.10	1.00	0.20	[64]

* Values for dung beetle is mg/100 g of dry weight basis.

### 2.4. Micronutrient and Vitamin Composition Variability

The composition of micronutrients and vitamins in insects depends on their dietary intake and exhibits variations across species, orders, and seasons. There are also significant variations in the literature accessible for particular species, which might be attributed to analysis at various phases. Insects often have adequate mineral levels to fulfill most animals’ nutritional needs. Insects with remarkably high levels of iron and zinc can offer substantial calcium, magnesium, manganese, phosphorus, and selenium. Despite the variable ranges of reported mineral content in insects, they consistently emerge as a valuable mineral resource. According to the Weru, Chege, and Kinyuru [40] review, phosphorus values ranged from 2.74 to 1443 mg/100 g, while magnesium varied from 1.54 to 1009.26 mg/100 g. This value for calcium was 0.27 mg/100 g in palm weevils at the late larval stage, while the highest value was for termites (*Trinervitermes germinates*). Similar trends in mineral content extend to other elements, with significant variations among diverse insect species. However, the majority of edible insects are particularly rich in iron. The amount of iron found in insects is generally greater than in fresh meat. This wide range in mineral content reported could be attributed to the source of the insects, whether wild or farmed, feeding source, etc. [39,65]. 

Insects exhibit notable variations in their vitamin composition. They are rich in B vitamins, such as riboflavin (B2), B5, and biotin, but have low levels of vitamin A, B3, niacin (B1), and vitamin D [38]. Previous studies also indicated minor quantities of vitamins in which vitamin E showed the highest levels of 0.925 mg/100 g for termite (*Nasutitermes* spp.), while the lowest reported value was for vitamin C at 0.0046 mg/100 g for termite soldiers (*M. bellicosus*) [55]. The vitamins and mineral content significantly depend on the insects and the source of feeds [52]. Compared to other species, yellow mealworms have the highest amount of vitamin B12.

## 3. Production of Protein, Protein Hydrolysate, and Peptides

The efficient extraction of proteins from insects, similar to protein extraction from other sources, needs a method that maximizes surface area exposure of the raw material to achieve optimal yields of both protein and fat/oils. In this sense, the insects should be dried first and then ground/sieved to obtain a homogenous insect flour. De-fatting the ground insect sample will facilitate the extraction of protein efficiently and proficiently. However, insect flour also could be used as a food ingredient for the formulation of different food products [66,67,68,69,70,71]; the European Commission also has recently approved the use of specific insect flours as food ingredients, in keeping with the need to identify alternative high-quality sources of protein for human nutrition [72]. The next step in protein extraction after de-fatting is the solubilization of protein. In order to separate the slurry and residue, centrifugation/filtration is typically used. The last steps to extract protein from insects as a protein source are the precipitation of protein and then the separation of the supernatant and protein sediment by another centrifugation procedure to produce protein concentrate/isolate from insects [38,40,42,67,73].

De-fatting the insect flour is a crucial step for extracting the protein because it improves the extraction yield and enhances the efficiency of the extraction process. It also prevents the interference of the fat/oil content with the protein and the problems resulting from interactions between the hydrophobic amino acids and fat, which appear to be the potential reasons for non-significant functional properties [24,67,71]. Different researchers have previously reported various solvents to de-fat insect flour or insect proteins in which hexane, ethanol, petroleum ether, and isopropanol were widely used as solvents [74,75].

Recently, there has also been the use of supercritical carbon dioxide (SC-CO_2_) extraction to recover lipids from insect powder and create protein-rich extracts for usage as food ingredients. SC-CO_2_ extraction has several advantages over conventional organic solvent extraction methods. As a solvent-free process, it reduces the potential for solvent residue contamination of the extracts. SC-CO_2_ extraction also helps to minimize the oxidation of unsaturated fatty acids compared to other methods. In a study performed by Kim et al. [76], they found SC-CO_2_ defatting of black soldier fly larvae powder for 6 h at 350 bar pressure reduced fat content below 5%. They concluded SC-CO_2_ extraction was an effective method to optimize the fat content of insect powder for feed production. In another study, Laroche et al. [77] compared SC-CO_2_ to various organic solvent extractions for mealworm and cricket powder defatting. For mealworm powder, SC-CO_2_ extraction yielded fat extraction (22%) similar to Soxhlet solvent methods, indicating its effectiveness. However, SC-CO_2_ extracted less fat from cricket powder (12%) compared to Soxhlet, suggesting effectiveness may vary by insect type. Overall, the studies demonstrate SC-CO_2_ insect powder defatting can achieve comparable or better results than conventional methods while offering sustainability benefits. Further optimization of parameters like temperature, pressure, and co-solvents could improve results.

The most widely applied method for protein solubilization from insects involves utilizing an alkaline pH environment. This method entails selecting an appropriate pH level and a specific solvent-to-insect ratio, determining the optimal reaction temperature and duration, and ensuring the mixture of insect flour and alkaline solvent are stirred to achieve maximal protein solubilization. In a study performed by Zhao, Vázquez-Gutiérrez, Johansson, Landberg, and Langton [74], NaOH was used to solubilize the protein. They also optimized the extraction condition to isolate the protein from yellow mealworm. After analysis of the data, the 0.25 M NaOH, Solvent: Insect ratio of 1:15, temperature of 40 °C, and time of 60 min were shown as the optimum conditions to isolate the protein. In another study, different extraction conditions were used in which the ratio, temperature, time, pH, and stirring speed were 1:25, 60 °C, 30 min, 11, and 300 rpm, respectively [67].

As reviewed by Villaseñor, Enriquez-Vara, Urías-Silva, and Mojica [71], different extraction conditions including a ratio of 6:1 to 20:1 (*v*/*w*), with pH ranging from 9.0 to 11.0, have been used to extract the protein from different insects. Zielińska, Karaś, and Baraniak [51] conducted a comparative analysis of the functional properties of three different edible insect species including tropical house cricket, desert locust, and mealworm. They extracted the protein using insect and 0.2% NaOH at a ratio of 1:10 (*w*/*v*) to maintain a pH of 11. The process was conducted at room temperature for 1 h. In another study, a ratio of 1:20 showed the highest protein solubility when different ratios were employed to extract the protein from the black soldier fly [78].

## 4. Hydrolysis of Insect Proteins

Recently, there has been growing interest in producing protein hydrolysates and peptides from various insect species, which is due to some issues related to insect proteins, including allergenicity and weak functional properties. As Zarei, et al. [79] explained, there are three different techniques of enzymatic hydrolysis of proteins based on how enzymes are added to the protein-containing buffer solution system. The methods are categorized into three different enzyme addition systems: multiple-enzyme digestion systems, simultaneous enzyme addition, and consecutive enzyme addition systems. In the single-addition systems, only one enzyme is added to the protein substrate, and hydrolysis will be carried out based on the respective enzyme. However, more than one enzyme (≥2) is added in the multiple-enzyme digestion and consecutive enzyme addition. According to the literature, most of the studies on enzymatic protein hydrolysis used the single-enzyme addition method.

In their study, Leni, et al. [80] used the protease derived from *Bacillus licheniformis* to facilitate protein extraction and improve the techno-functional properties of proteolysate from lesser mealworms. They also investigated the effect of the degree of hydrolysis (DH) on functional properties of the protein hydrolysate during a 3 h hydrolysis period. Their findings showed that the DH enhanced the solubility and oil-holding ability, whereas a reduced emulsifying ability was observed. Notably, the use of protease-assisted extraction has been applied by researchers worldwide. In a study carried out by Leni, et al. [81], six different proteases were used to hydrolyze the protein and assist the protein extraction from two species, namely the litter beetle and black soldier fly. They calculated the extraction yield and evaluated the degree of hydrolysis and the free amino acids. Their results demonstrated that the protein hydrolysates generated from the black soldier fly revealed a degree of hydrolysis ranging from 3 to 18%, while the hydrolysates from the litter beetle showed a DH between 7 and 23%.

Mintah, et al. [82] used an edible insect, black soldier fly larvae, as a protein source for hydrolysate production. This involved three distinct pretreatment methods: conventional, fixed-frequency ultrasonic, and sweep-frequency. The hydrolysis conditions using alkaline protease, characterized by a temperature of 90 °C, a hydrolysis duration of 90 min, and a pH of 9, were conducted under impeller agitation at 100 rpm. Their results indicated that ultrasound-assisted pre-treatment showed higher antioxidant activities, solubility, and foam expansion across a pH range of 2 to 12. Sousa, Borges, and Pintado [83] evaluated the different conditions to produce proteolysate from the litter beetle. The alcalaes to and corolase PP were used to hydrolyze the protein in different enzymes to substrate E/S ratios. Ultimately, their study identified the optimal conditions to be determined: a substrate ratio of 1.5% for 4 h and a ratio of 3% for 6 h. They also evaluated the ACE inhibitory, antioxidant, anti-diabetic, and antimicrobial activities, indicating no antimicrobial or antidiabetic properties. Yoon, et al. [84] extracted the protein from three insects including mealworm larvae, adult crickets, and silkworm pupae and then hydrolyzed it using flavourzyme and alcalase. The protein hydrolysates showed a higher solubility value, while the foaming capacity was lower in comparison to the control.

Furthermore, in a study performed by Vercruysse, et al. [85], the proteins of four insects, including cotton leafworm (*Spodoptera littoralis*), silkworm/domestic silk moth, desert locust, and buff-tailed bumblebee or large earth bumblebee (*Bombus terrestris*), were hydrolyzed using three proteolytic enzymes including gastrointestinal proteases, alcalase, and thermolysin. Although hydrolysis of the insect proteins resulted in increased ACE inhibitory activity, the highest ACE inhibitory activity was observed after gastrointestinal digestion.

Another insect sample used for generating protein hydrolysate was black soldier fly larvae. Prior to hydrolysis, the sample underwent a defatting process. The hydrolysis process was performed using the bromelain enzyme under conditions with a temperature of 50 °C, a rotation speed of 150 rpm, and a pH range of 6 to 8. Various hydrolysis duration ranged from 3 h to 24 h and different enzyme concentrations (1–5%) were applied, and the analysis results are shown in Table 6 [86].

Subcritical water has become a green, quick, and effective technology for protein hydrolysis as a substitution for the extensively used chemical, enzymatic, and fermentation procedures. In a study by Bae and Lee [87], they evaluated the impact of citric acid and sodium bicarbonate on the hydrolysis of subcritical water. To assess the temperature, pressure, and modifier concentration, researchers used a full factorial design. The hydrolysis of a protein concentrate produced a degree of hydrolysis (DH) of 38.73% with citric acid and 44.56% with 1 M sodium bicarbonate at 130 °C/20 MPa. Interestingly, treatments with citric acid showed increased bioactivities: IC50 values for DPPH, ABTS, and ACE inhibition were 4.06 mg/mL, 330 µg/mL, and 0.148 mg/mL, respectively.

**Table 6:** Different conditions and enzymes used for hydrolysis of various insects’ proteins.

	Insect	Protease(s)	Enzyme/Substrate Ratio	Temperature (°C)	Time	pH	Speed (rpm)	Reference
1	Tropical house cricket (*Gryllodes sigillatus*)	Alcalase	0.5–3.0%	50 °C	0.5–1.5 h	8.0	-	[88]
2	Mealworm larva (*Tenebrio molitor* (L.))	Alcalase	1:100	50 °C	4.0 h (250 min)	8.5.		[89]
3	Silkworm pupae (*Bombyx mori*)	Alcalase^®^ Prolyve^®^ Flavourzyme^®^ Brewers Clarex^®^	1% (*w*/*v*)	50 °C	4.0 h	8.0		[90]
4	Tropical house cricket (*Gryllodes sigillatus*)	Alcalase	3.0%	55 °C	80 min	8.0		[91]
5	Black crickets (*G. assimilis*)	FlavourzymeTM, Alcalase Neutrase	100 mg/mL	50 °C	4.0 h	7.0	100	[92]
6	Mealworm (*Tenebrio molitor*)	Alcalase^®^ Pepsin	0.03% (*w*/*w*) 0.25%	60 °C 40 °C	2.0 h 4.0 h	8.5 2.0		[93]
7	Lesser mealworm (*Alphitobius diaperinus* L.)	Enzymatic extract of mature artichoke flower	1% (*w*/*v*)	50 °C	16.0 h	6.2		[94]
8	Mealworm larvae (*Tenebrio molitor*), Criket (*Gryllus bimaculatus*), Silkworm pupae (*Bombyx mori*)	Flavourzyme Alcalase Neutrase Protamex	30 and 60 U/g protein 12 and 72 mU/g protein 4 and 24 mU/g protein 7.5 and 45 mU/g protein	55 °C	8.0 h			[84]
9	Lesser mealworm (*Alphitobius diaperinus*)	Alcalase Corolase	0.5–3.0% 0.5–3.0%	50 °C	0.0–24.0 h	8		[83]
10	Lesser mealworm (*Alphitobius diaperinus*) Black soldier fly larvae (*Hermetia illucens*)	Papain Pancreatin Dispase I Pepsin Protease From *Bacillus Licheniformis* Bromelain Trypsin	1:10	60 °C 37 °C 37 °C 37 °C 60 °C 50 °C 37 °C	18.0 h	6.5 7.8 7.3 3.0 7.5 7.0 7.8		[80]
11	White-spotted flower chafer (*Protaetia brevitarsis*)	Subcritical water	---	100–300 °C		---	---	[87]

## 5. Bioactive Peptides

Once the structure, characteristics, and functions of a specific insect protein are comprehended, chemical synthesis emerges as a valuable technique for producing and researching the desired peptide. Insect protein extraction for human food holds the potential as a practical customer acceptance approach. The development, recognition, and accomplishments associated with a particular insect peptide with bioactive effects such as antioxidants, antimicrobials, and antihypertensives could catalyze increased investment and expanded research into the extraction and supplementation of insect proteins [9].

As shown in Table 7, different enzymes have been used for the enzymatic hydrolysis of insect proteins [9]. The simulation of the human gastrointestinal digestive system with pepsin, trypsin, and R-chymotrypsin has also been studied. These investigations have demonstrated the ACE inhibitory effects of insect proteins, suggesting that silkworm/domestic silk moth hydrolysates revealed 100% ACE inhibitory activity while the parent protein (un-hydrolyzed protein) showed 50% ACE inhibitory activity. The in vitro ACE inhibitory activity of small peptides was found using protein from cotton leafworm edible insects. After fractionation in two steps using RP-HPLC and gel filtration, a tripeptide, Ala-Val-Phe, was identified and sequenced. The in vitro ACE inhibitory activity of the peptide showed an IC50 value of 2123 μM [95].

In a study reported by Dai, Ma, Luo and Yin [89], mealworm larva was used as the protein source. After hydrolysis by alcalase and fractionation using RP-HPLC and gel filtration, the peptide sequence Tyr–Ala–Asn was identified by tandem mass spectrometry with an IC50 value for Angiotensin I-converting enzyme (ACE) inhibitory of 0.017 mg/mL. Their findings indicated that mealworm larva protein could potentially be a viable candidate for incorporation within the food industry and nutraceuticals.

Cermeño, Bascón, Amigo-Benavent, Felix, and FitzGerald [90] hydrolyzed the silkworm pupae using Alcalase^®^, Prolyve^®^, Flavourzyme^®^, and Brewers Clarex^®^ proteolytic and evaluated the antioxidant activity of the hydrolysates. Based on their results, Alcalase and Prolyve hydrolysates revealed the highest scavenging activities. Subsequent to the identification, sequencing, and synthesizing of the peptides, peptides SWFVTPF and NDVLFF showed the highest antioxidant activity. Furthermore, in a study focused on the sequencing and identification of effective antihypertensive, anti-glycemic, and anti-inflammatory peptides derived from the in vitro gastrointestinal digests of alcalase-generated protein hydrolysates, a total of 28 peptide sequences were identified. Three peptides, YKPRP, PHGAP, and VGPPQ, were chosen for the molecular docking studies. Among these peptides, PHGAP and VGPPQ exhibited a higher degree of non-covalent interactions with the enzyme active site residues and binding energies comparable to captopril [91].

The antimicrobial effect of insect peptides has also been studied. Van Moll, et al. [96] evaluated the antimicrobial activity of peptides from 36 black soldier flies in various concentrations against different human pathogens including *Staphylococcus aureus*, *Escherichia coli*, *Pseudomonas aeruginosa*, *Candida albicans*, and *Aspergillus fumigatus*. The study incorporated a human cell line (MRC5-SV2) and positive controls as reference. The findings showed strong activity against Gram-negative test strains at low micromolar concentrations except for Hill-Cec6.

The bioactivity of two cecropins, namely Hill-Cec1 and Hill-Cec10, was further investigated by characterizing their hemolysis, time-to-kill kinetics, membrane-permeabilization properties, and anti-biofilm activity. Hill-Cec1 and Hill-Cec10 showed high bioactivity against other bacterial species, including *Klebsiella pneumoniae* and multi-drug-resistant *P. aeruginosa*. Both antimicrobial peptides demonstrated bactericidal effects and had a rapid onset of action with membrane-permeabilizing effects. Hill-Cec1 and Hill-Cec10 were also able to prevent *P. aeruginosa* biofilm formation, although their impact on biofilm eradication was less pronounced. Overall, Hill-Cec1 and Hill-Cec10 are promising leads for the development of novel antimicrobial agents to treat critical infections caused by Gram-negative pathogens such as *P. aeruginosa*.

## 6. Bioactive Peptides from Insect Proteins: Functional Potential and Applications

The functional properties of insect proteins, including their protein concentrates, isolates, and hydrolysates, are governed by the processing methods employed during their extraction from insects. Based on the techno-functional properties of the protein, protein hydrolysate, and peptides, which are mostly dependent on the protein extraction method, amino acid composition, side groups of amino acids, protein/peptide chain length, and structure conformation, proteins create complex systems when interacting with another protein or other compounds such as lipids, carbohydrates, and minerals. Therefore, the functional characteristics of food products cannot solely be attributed to their protein content because other nutrients and compounds also play an important role in these characteristics [101].

### 6.1. Protein Solubility

According to Gravel and Doyen [101], solubility is one of the main functional characteristics in food systems, as many other functional features such as foaming, gelling, and emulsions are affected by the amount in which the protein can dissolve into an aqueous solution. The solubility of proteins depends on a number of intrinsic parameters such as the amino acid composition and structure, protein size, and 3D structure, and external factors like ionic strength, pH, and temperature. Effectively, their solubility is determined by the interaction between proteins and water. Negatively charged and polar amino acids improve solubility on the protein surface, while non-polar amino acids lower solubility.

Zielińska, Karaś, and Baraniak [69] studied the functional properties of proteins isolated from three species of edible insects including the tropical house cricket, desert locust, and mealworm, and evaluated the solubility of the isolated proteins. Their research found that the proteins exhibited their lowest solubility at a pH level of approximately 5 across all three protein samples, while the highest solubility was achieved at around pH 11. A high protein solubility was also shown within the pH range of 2 to 4. Similar results have been reported for larvae of an edible insect, the black soldier fly, in which the lowest solubility was shown at pH values of 4 to 5. However, the solubility increased beyond this range [78]. Mexican fruit fly protein also showed the minimum protein solubility at pH 5 whereas its maximum solubility was at pH 10 [66]. Furthermore, the solubility of proteins from desert locusts and European honeybees also varied from a minimum pH of 4 for desert locust and pH of 5 for European honeybees [102].

### 6.2. Edible Insect Proteins as Emulsifiers

Emulsions represent a homogeneous combination of two immiscible liquids, including oil droplets in water or water droplets in oil. The amphiphilic nature of protein facilitates the formation and stabilization of food emulsions by reducing the surface tension at the oil–water interface. This feature has numerous practical applications, including baked products, mayonnaise, salad dressing, frozen desserts, and comminuted meats. Conventional proteins such as milk, egg, and soy have been widely utilized as additions due to their emulsifying characteristics. However, recent research has delved into the emulsion characteristics of the most popular edible insect proteins. Many studies demonstrate that proteins obtained from edible insect sources could be potentially used in food composition under appropriate pH and ionic strength conditions [73,101].

Zielińska, Karaś, and Baraniak [69] compared the emulsifying properties of three insect species in which the highest value was noted in the tropical house cricket protein preparation (72.62%) with an emulsion stability of 38.3%. Similarly, Omotoso [103] reported an emulsion activity of 75% for silkworms but with a lower emulsion stability (23%). The emulsion activities for the whole insects in the Zielińska, Baraniak, Karaś, Rybczyńska, and Jakubczyk [58] study were found to have consistent values ranging from 62% to 69.17%. Moreover, pretreatment with pulsed-field electricity (PFE) could improve the emulsifying capacity (EC) of proteins extracted from the house cricket flour. The highest intense PFE increased the emulsifying capacity to 74.7%, while the lowest increase in the EC was 22.1%.

Emulsifying properties, including the emulsifying capacity (EC) and emulsion stability (ES), of proteins extracted from the grasshopper desert locus and honeybee were also studied [102]. Results indicated that the highest emulsifying capacity (EC) was shown for protein fractions of the grasshopper desert locus and honeybee. However, the highest emulsion stability (ES) was observed for protein fractions from the grasshopper desert locus. Table 8 exhibits the functional properties of protein from some edible insects as well as other protein sources, including plant and animal sources.

### 6.3. Foaming Properties of Insect Proteins

Foams are described as air bubbles in a liquid that are stabilized by proteins at the air–liquid interface. Similar to functional characteristics, foam properties are governed by a range of factors, including protein structure, amino acid composition, protein chain length, etc. As foam production requires protein unfolding to improve air-water adsorption, globular and compact proteins generally have less efficacy in foam formation compared to their fibrous and elastic counterparts. In contrast, the rapid production of air bubbles does not inherently correlate with foam stability, which is essential in the formulation of food products. Highly interconnected and densely structured globular proteins create more robust films, enhancing their resistance to deformation compared to flexible proteins. This property also contributes to their ability to produce more stable foams. In contrast to the emulsifying effects that can be improved by the presence of hydrophobic amino acids on the surface of the protein and the general presence of oil in the protein sample, the presence of fat tends to affect the spraying effects. Defatted proteins were reported for a higher spraying capacity and stability compared to non-defatted protein meals. Furthermore, the hydrolysis of proteins has a beneficial influence on moisturizing qualities, potentially by increasing protein flexibility; this improves the adsorption rate of the peptides produced on the interface and raises the initial foam capacity.

Egg-white proteins are the most widely utilized proteins in spray applications. Furthermore, milk proteins such as whey proteins and caseins or soy proteins are also utilized for spray utilization. The characteristics of foam inherent to these proteins have been investigated, especially within the most popular edible insect proteins. These protein sources exhibit potential for integration into food formulations for foaming purposes under specific pH and ionic strength conditions [101]. The foaming activity and stability are different among various insects, even when subjected to a similar processing approach. One of the main factors affecting the foaming properties is the amino acid composition of insect proteins, as shown in Kim, et al. [107]. In that study, the salt-soluble protein fraction of mealworms revealed significantly higher foamability than water-soluble fractions. Having a polar water-soluble group (amphiphilicity) is a critical parameter in foaming properties. No foam activity was observed in the African migratory locust flours at a pH of less than 3, while a significant foam formation of around 200% was shown at pH 5 [108].

### 6.4. Gelling Properties of Insect Proteins

Protein gels need to be clearly described as their definition depends on the employed characterization technique. For example, gels may be defined as an organized network of proteins capable of retaining a significant amount of water and showing no stagnant laminar flow. As with every other functionality mentioned above, gelation depends on several inherent elements, such as electrostatic interactions and external factors like temperature, which could be considered the key factor. Within the formation process of gel, heat is crucial because it can accelerate rapid protein development and denaturation, causing delayed rearrangement and aggregation and producing the ideal gel throughout the refreshing process.

Limited research has been conducted to identify the gelling characteristics of edible insect proteins. Yi, et al. [109] investigated the gel-forming capacity of three distinct orders of five insect proteins, including mealworm, darkling beetle (*Zophobas morio*), lesser mealworm, house cricket, and dubia cockroach (*Blaptica dubia*) (Coleoptera, Orthoptera, and Blattodea). It was concluded that gels from all concentrations might be made from pH 7 or 10 at a protein concentration of 30% (*w*/*v*). However, the soluble fraction of the house cricket formed a gel at pH 7 and a concentration of 3% when it was heated at 86 °C. The gelling property of an insect’s protein concentrate is influenced by enzymatic hydrolysis. Dion-Poulin, et al. [110] indicated that enzymatic hydrolysis has a negative impact on gelation in the protein hydrolysates produced from cricket and mealworm proteins.

### 6.5. Water-Holding Capacity of Insect Proteins

All conditions associated with the protein matrix’s capacity to retain the maximum amount of water per gram of the sample material, even when subjected to the force of gravity, are called the water-holding capacity (WHC). Water-holding capacity (WHC), water-binding capacity (WBC), and water absorption capacity (WAC) are all the same terms used to elucidate how a protein is able to absorb water. Despite potential variations in measurement methodologies, these three terminologies are commonly employed interchangeably. This functional characteristic is indeed related to the gelation and gelling properties of proteins. Furthermore, the water-binding capacity is enhanced through thermal denaturation. This feature is also linked to a better texture and wetness that is of considerable relevance in food formulation. Certain insects like mealworm and African palm weevil (*Rhynchophorus phoenicis*) have greater WHC values than pulse protein flours and are similar to concentrated soy and milk protein levels. These interesting results show that specific concentrated insect protein or flour may be included as functional agents in food formulation [101]. In a study, the functional properties of three species of edible insects, including the tropical house cricket, desert locust, and mealworm, were investigated. The results indicated that the highest water-holding capacity was noticeable for the mealworm at a 3.95 g/g protein concentration, almost comparable with soy and mung bean proteins with 3.00 and 3.33 g/g, respectively [104].

### 6.6. Oil Absorption Capacity of Insect Proteins

The capacity of oil absorption (OAC), the capability to bind to oil (OBC), and the capability to maintain oil (OHC) all refer to the number of lipids absorbable by the amount of protein powder indicated. These functional property terms are significantly linked. Hydrophobic proteins and small, low-density proteins exhibit heightened lipid affinity compared to large, high-density, and hydrophilic equivalents. As protein contains different hydrophobic amino acids that have hydrophobic properties, it can interact with oil in foods. In fact, the water and oil absorption capacity depends on the availability of polar and non-polar amino acids. Less availability of polar amino acids correlates with reduced water absorption capability, and vice versa [104]. The oil absorption capacity values for black soldier fly protein obtained by a modified protocol (MP) have been evaluated using a modified protocol (MP) with conditions of 60 min, a 15:1 alkaline-solution-to-sample ratio, and 40 °C. Results showed comparable values with the tropical house cricket (3.22 g/g) and the desert locust (3.22 g/g) protein extracts [78], while that for the optimized protocol (OP) was reported to be around 2.74 g/g for mealworm. These results showed a higher value for OAC compared to the 1.08 g/g reported for the protein extract from the plant seed source [111].

## 7. Edible Insects and Their Proteins in Meat Analogs and Cereal Products

According to Gravel and Doyen [101], several studies have shown the inclusion of insect proteins and analogs in beef emulsions. De-fatted mealworm flour, or protein hydrolysate, has substituted 10% lean pork in emulsified sausages [112]. The meat also hardened compared to the control sausage, irrespective of the initial preparation. The added protein-rich insect meal resulted in considerable moisture loss in the emulsified sausage, thereby changing the texture. Similar research with the house cricket was carried out by the same authors, and the conclusion was that insect proteins might strengthen emulsified meats. Smetana, et al. [113] explored the replacement of a fraction of soy content with lesser mealworm protein concentrate and succeeded in recreating the texture and humidity of a meat analog.

Moreover, in recent years, there has been growing interest in the incorporation of insect protein into various food products, including pasta, bread, and other pastries. In a study performed by Azzollini, et al. [114], a cereal snack incorporated with 10% and 20% mealworm flour was developed, and a comprehensive evaluation encompassing nutritional, physical, and microstructural aspects was undertaken. The results revealed that the addition of insect flour enhanced protein content and snack digestibility. The 10% mealworm flour cereal snacks displayed similar textural features as their insectless counterparts. However, those with 20% mealworm flour showed poor structural characteristics. The authors emphasized that the important stage during the formulation of protein-rich dietary products is when selecting the processing method, as the texture and nutritional characteristics may differ appropriately. Cuj-Laines, et al. [115] have studied the addition of various amounts of grasshopper (*Shenarium purpurascens*) flour in extruded maize snacks. A sensory assessment panel was added to their study, and snacks with the lowest grasshopper percentage were chosen. The authors assumed that the brownish color and distinctive taste of grasshopper flour negatively influenced the consumer’s acceptability and advised the masking of other additives. However, specific strategies for implementation were not further elucidated in the study.

In another study, Djouadi, et al. [116] formulated a protein-rich healthy cracker from insect flour using 2% to 20% mealworm flour content and evaluated the nutritional, physical, and sensory properties of the crackers. The results showed an increase of 15% in protein content and an enrichment in minerals, including potassium, phosphorus, copper, and zinc. However, a darkening of the samples with the increase in the incorporation of mealworm flour was observed. However, the crackers containing 6% insect flour were the most accepted by the panelists.

Pasta preparation from insects also has been another area of food processing and development. Duda, et al. [117] tested the use of 5% cricket powder in wheat pasta. The authors investigated the effects of insect proteins on cooking time, color, texture, and flavor, with the latter being the most distinguishing feature. In general, the sensory assessments revealed that fortified wheat pasta meets consumer needs, showing no significant differences from other wheat pasta.

House cricket and yellow mealworm are beneficial insects, and their potential use for pasta formulation has been studied by Pasini, et al. [118]. In this study, the authors replaced semolina with 14% protein. Through a comprehensive assessment of technological quality metrics, the integration of insect-derived protein into the pasta resulted in a darker and firmer product compared to the control. In general, adding insect protein to the formulations enhanced the quality and protein content of the pasta product.

Similar to the utilization of insects and insect proteins to fortify pasta products, the incorporation of insects and/or insect proteins into bread formulations has also been explored. Osimani, et al. [119] combined wheat flour with cricket powder at proportions of 10 or 30% to enhance bread quality. While the addition of cricket flour increased the nutritional value, particularly in terms of protein, it also adversely impacted the hardness of the loaf. The researchers noticed a linear association in the integration of insect meal with an increased volume and hardness. Nonetheless, bread enriched with 10% of the house cricket indicated a discrete global liking by untrained panelists.

Additionally, in a study conducted by González, et al. [120], the evaluation of incorporating insect-derived flours derived from black soldier fly, house cricket, and mealworm into bread dough was undertaken. The authors concluded that the house cricket was the most suitable flour for bread formulation as it had the best functional properties and flavor of the three insects. The authors also noted the necessity for further research endeavors to align with consumer expectations and high standards [101]. In another study by Ribeiro et al. [121] the majority of participants were unwilling to consume bread that had been enriched with edible insects. However, specific sorts of bread that included processed insects were more widely accepted. Rejection of eating insects was highly correlated with disgust for them.

Kowalski, et al. [122] also evaluated the impact of insect flour supplementation on bread quality and nutritional values. The addition of 10% insect flour significantly increased the protein content compared to wheat bread. Furthermore, the amino acid score (AAS), particularly for lysine, increased from over 40% to approximately 70%. Sensory analysis of samples also showed that the addition of edible insects flour to bakery products is generally acceptable, at up to 10% of supplementation.

## 8. Challenges, Food Safety, and Considerations in Utilizing Edible Insects and Their Proteins as Food Ingredients

**Figure 2:**
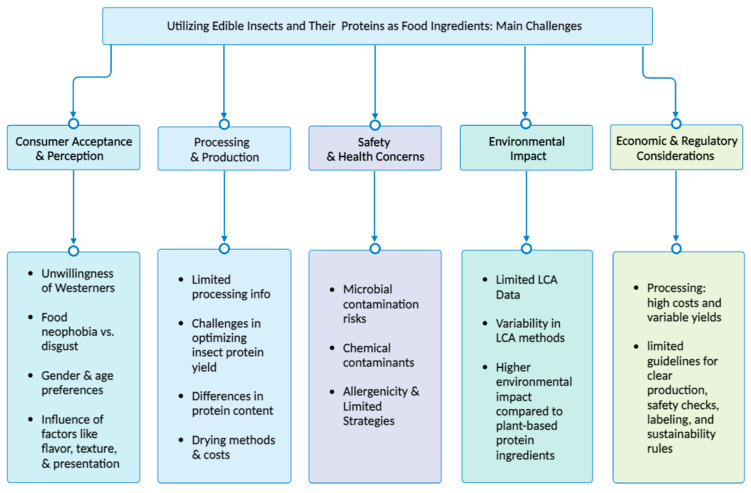
The main challenges of utilizing edible insects and their proteins as food ingredients.

Although the consumption of insects, insect flours, and their protein isolates and concentrates is increasing worldwide, there are still some issues and challenges that need to be considered and addressed by researchers, producers, manufacturers, and companies. Among the paramount challenges associated with insect entomophagy and the integration of insect-based flour or protein into various products, several pivotal concerns demand consideration, including consumer acceptance, processing methods, environmental impact, economic considerations, the imperative for regulatory frameworks and legislation, and the assurance of food safety to both insects and their derivative products. The detailed considerations delineated in the subsequent sections are succinctly encapsulated in Figure 2.

The food safety concerns that can cause health problems in consumers include potential allergenicity, toxicity due to chemical contamination, and the risk of microbial contamination. Allergenicity is a critical issue and a significant challenge that needs to be carefully addressed in all research and insect-related feed and food industries. Allergic foods, particularly shellfish (mostly shrimp, lobsters, and crayfish), are common inducers of allergic responses among susceptible individuals who might be allergic to foods. Extensive literature surveys consistently identify tropomyosin and/or arginine kinase as the major proteins responsible for allergic reactions, resulting in patients with crustaceans and/or house dust mite allergies being more prone to also being allergic to edible insects; however, more information about the molecular mechanisms underlying food allergy to insects is needed. Although some studies focused on reducing or eliminating allergens in insects and their derivative products, only a few food-technology procedures, such as fermentation and hydrolysis, might significantly reduce food allergy [123,124].

Another big concern is chemical contaminants, including heavy metals such as cadmium, mercury, lead, and arsenic, and accumulated environmental contaminants, such as hormones and pesticides. van der Fels-Klerx et al. [125] reported that cadmium and arsenic were found in two important insects used in the human diet (black soldier fly and yellow mealworm larvae). Factors that could increase the presence of chemicals and metals are the insect species, growth phase, and feed source. The presence of pesticides is a concern when insects are harvested from the wild, for example, in a rice field where pesticides have been applied to control insects and protect the crop. However, these chemical and pesticide concerns can be reduced by good production and pre- and post-harvesting practices, in which factors such as feed and species can be controlled [126].

In addition, microbial contamination, both spoilage and pathogen microorganisms, is a concern when using insects as a food source. Insects could serve as vectors for pathogens like *Salmonella* spp. and *Campylobacter* spp. or harbor spore-forming bacteria in insect-related products, potentially facilitating human transmission [127]. However, this is a concern when insects are consumed raw as ready-to-eat food. Kolakowski et al. [128] studied the presence of *Salmonella* spp. and *E. coli* in two different insects’ (cricket and silkworm) flours, powders, bars, and whole insects and could not detect these pathogens. An explanation for this is that the adequate drying, steaming, or cooking process can help control pathogens in the final product. In addition to that, proper handling of these products will help keep the product safe [126]. Parasites are another biological hazard related to some specific insect species. Boye et al. [129] reported that parasites such as *Entamoeba histolytica*, *Giardia lamblia*, and *Toxoplasma* spp. had been isolated from insects such as cockroaches. This hazard still needs to be studied more in depth, and to reiterate, it has been associated with insects harvested from wild habitats. Nonetheless, the main challenge with the consumption and incorporation of insects in the human diet is the public resistance to trying this product and its derivates.

As reviewed by Gravel and Doyen [101], the unwillingness of Westerners to try edible insects is one of the most significant difficulties in integrating them into foodstuffs. Although insects are sold as a delicacy in certain experimental restaurants, eating them is repulsive for most consumers. A few research studies have shown the two main obstacles to ingestion, namely food neophobia and disgust, with the latter exerting a more pronounced influence. These researchers also indicated that enhancing the overall entomophagy factors, such as flavor, texture, and presentation, holds considerably more promise in engendering the acceptance of insects as a proteinaceous alternative, in contrast to solely fostering familiarity to mitigate food neophobia [130]. Although most researchers agree that food neophobia is important for the consumer’s hesitation to try insects, the distinction between food neophobia and disgust is not quite apparent [101].

Several studies have indicated that individuals inclined to consume insect-based food products tend to exhibit a higher appetite for adventurous behavior. Investigations have additionally revealed a gender-based disparity, with males displaying a greater propensity to accept insects as a substitute for conventional protein sources because they are described, on average, as more adventurous eaters. Younger consumers experienced fewer adverse effects from consuming insects compared to older consumers. These studies also recognized the value of producing goods with transformed insects since insects were not visible to consumers and were made more appealing [101].

Another concern is the information and knowledge on processing techniques for optimizing insect protein yields and purity, which remains sparse and typically takes place solely on a laboratory scale, posing a significant challenge in insect protein processing. As previously noted, insect proteins are generally obtained using already recognized and utilized techniques for herbal goods. The protein content and functioning data vary greatly from insect species to ways of breeding and processing. Therefore, future research is needed to identify optimum processing conditions in order to produce food-based insect protein isolates with high functional characteristics, cost-effectiveness, and environmental sustainability.

Another issue is the drying of insects and insect-based ingredients in which non-denaturing drying techniques for laboratory investigations, such as freeze-drying, supercritical carbon dioxide, and microwave drying, are too expensive for commercial usage versus conventional thermal drying. Since using a specific drying method may affect the functional properties of some insects, using a combination of techniques could be a more efficient approach to dry the insects and insects’ proteins.

In terms of environmental impact, the initial focus on insects as a novel protein source in the fields of agriculture and food science was prompted by the Food and Agriculture Organization in 2013. This research underlines that insect farming uses less space, water, and feed, making it more ecological than traditional animals. As the insects are cool in blood, they do not have to expend as much energy to maintain a constant temperature and, therefore less food to maintain homeostasis [131]. This also impacts its emission and conversion of greenhouse gases. Oonincx, et al. [132] assessed the greenhouse gas (GHG) emission levels of five distinct insect species in their research and determined that four of the five contributed barely 1% of the ruminants’ GHG.

The researchers also examined the emission of ammonia from these insects and discovered that all values were below the normal animal. The authors evaluated the feed conversion ratio (FCR) across four insect species. Among these species, the dubia cockroach and black soldier fly were more effective in converting their feed than traditional livestock, whereas mealworm and house cricket had FCRs equivalent to pigs and poultry. A more comprehensive assessment of the environmental impact of a food source across the production chain is performed by the Life Cycle Assessment (LCA). However, few LCAs for insects were published. Halloran, et al. [133] emphasized the necessity of establishing precise standards for future research, enabling a clear insect evaluation as an alternative to sustainable foods. The different methodologies led to incomparable results and varying conclusions, depending on the functional unit and global aim of each study. In general, while concentrated insect-derived protein had a lower environmental footprint compared to animal-based protein concentrates, they exhibited a higher environmental impact than plant-based protein ingredients.

In view of previous studies, it is evident that using insects from safe and suitable food sources, coupled with the processing procedures that minimize the risks, is essential for insect applications as possible sources of protein and other nutrients [101,134].

## 9. Halal and Kosher Considerations for Insect-Based Food Products

For most Muslims, their food acceptability on a global scale is firmly dependent on religious rules, which are monitored and verified by the Halal certification bodies, especially for the food produced by companies. Based on the International Market Analysis Research and Consulting (IMARC) Group report entitled “Halal Food Market: Global Industry Trends, Share, Size, Growth, Opportunity, and Forecast 2018–2023”, the global halal food market reached a value of US$1.4 trillion in 2017. The report projected the market value to reach US$2.6 trillion by 2023, exhibiting a compound annual growth rate (CAGR) of more than 11 percent during the period between 2018 and 2023. With the growing number of Muslims worldwide and the continued agitation for healthier and trusted food products, the halal food market has emerged as one of the most profitable and influential markets in the contemporary business world [135].

However, the Islamic Canon law shows different and mostly opposing views regarding insects and their products, which mainly depend on the various Islamic branches and opinions. An example is Carmine (E-120), an insect extract that is used as a food dye in the food industry. However, Islamic scholars have different opposing views about its usage in food products. Therefore, these opposing views have significantly affected Islamic scholars, followed by the certification bodies.

Although there is ambiguity and obscurity among the Islamic scholars and branches, the Halal logo is not only a religious indication or symbol for only a specific population now. It has become a symbol of clean, hygienic, and reliable food products, acknowledged by a majority of Muslims and some non-Muslims. [135]. Therefore, it would be a contradiction when ignoring the various benefits of insect-based products, including protein, protein hydrolysates, or bioactive peptides. In addition, a general rule in Islam dictates that “All foods are considered Halal until proven to be Haram”. Thus, when Islamic scholars have different opinions about insects and the consumption of their products, there should be a critical examination and analysis of the scholar’s reviews and their evidence to verify the opinions’ validity.

In conclusion, although insects and insect-based products are not absolutely allowed or prohibited in Islam for consumption, forbidding the consumption of all insects as Haram is also a simplification and generalization of the opinion. This is evident in the consensus of the four Sunni schools and most Shia Scholars that locusts and food worms that grow out of it are halal. However, there has yet to be a consensus among them on other edible insects. Based on the juristic analysis, the opinion that allows insect consumption under some conditions is considered more evident and preponderant. However, an essential precondition before certifying an insect-based food product as halal is an evaluation that encompasses a risk analysis as well as the nutritional quality of each insect that is being marketed. It is then left to the consumer to choose such food items as a matter of individual acceptability, but such food items should not be denied halal certification [135].

According to Kosher dietary laws, the consumption of crustaceans and almost all insects is prohibited. Consequently, substances like carmine and cochineal, which are used as natural red pigments, are not permitted in kosher products by most rabbinical supervisors. The exception includes a few types of grasshoppers, which are acceptable in regions where the tradition of their consumption has endured. The edible insects are all in the “grasshopper” family identified as permitted in the Torah due to their unique “jumping” movement mechanism. Again, only visible insects are of concern; an insect that spends its entire life cycle inside a single food is not of concern. Recent advancements in exhaustive cleaning methods for prepackaged salad vegetables have substantially reduced the insects that are sometimes visible, rendering the product kosher-certified. This certification ensures the viability of these products both in kosher food service establishments and private homes, eliminating the necessity for extensive specialized inspection procedures. Although significant efforts are invested by companies to produce an insect-free product, some kosher supervision agencies remain unconvinced. They only certify those products that meet their more stringent requirements or particular production lots (for example, one day, the production may be acceptable and the next day it might not). The prohibition of insects focuses on the whole animal. In scenarios where the intention is to process food using a blender or food processor, inspection of fruits and vegetables for insects may be bypassed, with the assumption that the presence of insect fragments does not compromise the food’s kosher status. There are guidebooks describing which fruits and vegetables in particular countries need inspection and recommended methods for doing this inspection. Kosher consumers have found value in the use of pesticides to keep products insect-free, as well as the use of prepackaged vegetables that have been properly inspected. Modern integrated pest management programs that increase the level of insect infestation in fruits and vegetables can cause problems for the kosher consumer. Examples of unexpected insect presence include insects under the “triangles” on asparagus stalks, under the “greens” of strawberries, and thrips on cabbage leaves. Kosher consumers and “mashgichim” (religious supervisors on site) are trained to properly inspect those fruits and vegetables. Because of the difficulty of adequately inspecting them, many Orthodox consumers choose not to use Brussels sprouts [136].

## 10. Innovations and Contributions

Throughout this review, we have worked not only to synthesize the body of knowledge already available on using insects as valuable sources of protein and peptides but also to identify new relationships and critical perspectives that advance the discussion about sustainable protein sources in general. Our analysis of various insect species and the proteinaceous products derived from insects showed complex patterns and correlations that provide light on the potential of insects to address global food security and safety issues. This thorough investigation offers a valuable resource for upcoming research projects by laying the groundwork for comprehending the nutritional worth and functional characteristics of proteins and peptides derived from insects.

We have highlighted essential gaps in knowledge requiring more investigation by pointing out the weaknesses of the current literature. This study provides a roadmap for future studies, suggesting research directions that can advance the field in addition to being a compilation of present knowledge. Our review of the literature also considers its practical applications. The protein content of insects is comparable to that of traditional animal sources, ranging from 60% to 80%. This emphasizes the importance of insects as a sustainable and feasible alternative for human consumption. This realization is especially important in the context of the increasing concerns about the ethical issues and environmental impact of traditional protein sources.

By adding visual elements like tables and figures, we intended to improve the information’s comprehensibility and accessibility. These additions help to support significant findings and enable a more sophisticated understanding in addition to organizing complex data. To sum up, this review is more than just a summary of the material that has already been published; it also hopes to provide fresh viewpoints, methodological understandings, and valuable implications to the discourse around alternative protein sources. By synthesizing various studies, presenting critical reviews, and proposing suggestions for future research, we hope to encourage more study in this developing subject.

## 11. Research Limitations and Future Research Recommendations

Although our research offers useful knowledge on the potential of bioactive peptides and proteins derived from insects, it is important to acknowledge some inherent limitations in the field as a whole. One challenge is the variation in the protein and peptide content amongst insect species. There are significant variations in the nutritional profiles of different insects, notably in terms of protein content and peptide composition, due to their varied ecological environments, diets, and life stages.

Variability in reported findings results from the absence of uniform protocols for protein extraction and peptide identification that exist between studies. The lack of widely recognized methods makes it difficult to compare findings directly between studies and highlights the necessity of using standardized procedures in future studies.

In addition, the field’s dynamic nature necessitates continuous adaptation due to ongoing developments in technology, analytical techniques, and knowledge. Maintaining the relevance and accuracy of findings will need ongoing monitoring of advancing methodologies. Consumer acceptability of products produced using insects is still a major barrier. There is a great deal of variation in consumer attitudes, sensory characteristics, and cultural perspectives on consuming insects, all of which demand additional exploration.

In the future, studies should consider an in-depth investigation of different insect species, considering factors like regional variations, insect biology, and possible variances in protein content and bioactive peptides. Investigating cutting-edge methods for peptide identification and protein extraction could increase productivity and yield while advancing the development of sustainable and scalable procedures. It is critical to understand the sensory and cultural characteristics that impact consumer acceptance of insect-derived proteins in order to integrate them into mainstream diets successfully.

Furthermore, a crucial subject for further research is the creation of clear regulations and standards related to the use of proteins obtained from insects in food products. It is also crucial to evaluate the long-term health effects, including potential allergenicity, nutritional advantages, and effects on specific health conditions. The successful integration of insect-derived proteins into mainstream food systems would depend extensively on research on the economic viability of large-scale production, considering market dynamics, supply chain considerations, and cost-effective rearing techniques.

## 12. Conclusions

Edible insects are abundant in protein content, encompassing all essential amino acids required for a balanced diet. This is a viable alternative protein source for feeding options to the expanding world population. It has significant potential for enhancing food security, offering a sustainable and environmentally conscious alternative to conventional protein sources. Most of the available data derive from insect populations harvested in the wild, suggesting that insects in some orders are similar in protein composition to conventional proteins such as animal-based proteins and plant-based protein dietary sources such as soy, fish, and poultry.

The significance of insects as a potential source of human consumption arises from their protein content, comparable to or slightly lower than animal-based protein within the range of 60–80%. Meanwhile, the amount of protein content found in insects is higher than in plant-based protein, which is around 45% to 50%. The amount of fatty acid content found in insects is less than in animal-based protein but higher than in plant-based protein; hence, it is acceptable. The predominant fatty acid found in edible insects like mealworm and house cricket is 18:1 n9 (oleic acid) and 18:2 n6 (linoleic acid). This meets the fatty acid requirement since fish and soy are also rich in oleic acid. However, insects cannot beat animal-based protein like fish, which is rich in EPA and DHA. EPA and DHA have important biological functions in vertebrates, and it is necessary to include EPA and DHA in the diet. Many minerals such as iron, zinc, potassium, sodium, calcium, phosphorus, magnesium, manganese, and copper are also detected in insects. The protein and amino acid composition of edible insects are highly varied, partly due to the absence of dietary standards, insect gathering, and processing locations. In addition, the use of insect-derived protein represents current improvements in insect peptides as antihypertensive, antibacterial, and antioxidant agents. Furthermore, a viable biotechnology industry lies in the large-sized manufacturing of insect bioactive peptides.

In order to address the needs of worldwide society, a comprehensive approach is necessary to address the challenges, including consumer acceptance, economy viability, processing feasibility, and allergic hazards. Expanding insect production is crucial for achieving consistent quantity and quality, facilitating their large-scale integration. Meanwhile, it should lower the cost of insect rearing to compete with protein sources currently utilized. The primary goal of incorporating insects into mainstream food crops is to substitute existing protein sources that are considered overexploited and environmentally harmful. Achieving these objectives requires a multidisciplinary approach.

## Figures and Tables

**Table 1:** Environmental impact of farming on common protein sources.

	Insect	Chicken	Pig	Cow
Greenhouse gases released per kg of live weight, g	2	NA	1130	2850
Feed required per kg of live weight, kg	1.7	2.5	5	10
Land required per g of protein	18	51	63	254
Water required per g of protein, liter	23	34	57	112

Cadinu, Barra, Torre, Delogu and Madau [5], Aiello, et al. [6], Alexander, et al. [7], Agnieszka de Sousa [8].

**Table 2:** Edible insect species with a high potential for consumption.

Order	Vernacular Name	Scientific Name
Coleoptera	Lesser mealworm	*Alphitobius diaperinus*
Coleoptera	Black fungus beetle	*Alphitobius laevigatua*
Coleoptera	Palm weevil	*Rhynchophorus ferrugineus*
Coleoptera	Mealworm	*Tenebrio molitor*
Coleoptera	Giant mealworm	*Zophobas atratus*
Diptera	Blowfly	*Chrysomya chloropyga*
Diptera	Black soldier fly	*Hermetia illucens*
Diptera	Housefly	*Musca domestica*
Hymenoptera	European honeybee	*Apis mellifera*
Hymenoptera	Leafcutter ant	*Atta laevigata*
Lepidoptera	Lesser wax moth	*Achroia grisella*
Lepidoptera	Silkworm/domestic silk moth	*Bombyx mori*
Lepidoptera	Greater wax moth	*Galleria mellonella*
Lepidoptera	Mopane worm/caterpillar	*Gonimbrasia belina*
Orthoptera	House cricket	*Acheta domesticus*
Orthoptera	Tropical house cricket	*Gryllodes sigillatus*
Orthoptera	Jamaican field cricket	*Gryllus assimilis*
Orthoptera	Two-spotted cricket	*Gryllus bimaculatus*
Orthoptera	European field circket	*Gryllus campestris*
Orthoptera	African migratory locust	*Locusta migratoria migratorioides*
Orthoptera	Bush-cricket	*Mecopoda elongata*
Orthoptera	Grasshoppers	*Oxya* spp.
Orthoptera	Grasshoppers	*Melanoplus* spp.
Orthoptera	Grasshoppers	*Hieroglyphus* spp.
Orthoptera	Bombay locust	*Acrida* spp. *Patanga succincta*
Orthoptera	American grasshopper	*Schistocerca americana*
Orthoptera	Desert locust	*Schistocerca gregaria*
Orthoptera	Common cricket	*Teleogryllus mitratus*

Ordoñez-Araque and Egas-Montenegro [11] and Patel, Suleria and Rauf [12].

**Table 3:** Protein content of common and edible insects on a dry weight basis.

Insect’s Name	Protein Content Range (%)	Reference
Larvae	26–45	[27,28,29,30,31]
Cricket, Grasshoppers, Locusts	6–77	[27,28]
Grasshopper	20–56	[28,29]
Beetles, Grubes	8–69	[28,32,33]
Termites	20–43	[28,29,33]
Bees, Ants	5–66	[28,33]
Dragonfly	26–54	[34]
Cockroaches	43–66	[33]
Flies	35–64	[33]
True Bugs	27–71	[33]
Butterflies, Moths	18–60	[33]
Dragonflies, Damselflies	54–56	[33]

Values are expressed on dry dry-weight basis.

**Table 4:** Amino acid content comparison (mg/g protein) among various edible protein sources including insects, beef, and fish (on dry weight, mg/g protein).

Source	Lys *	His **	Arg **	Asp	Thr *	Ser	Glu	Pro	Gly	Ala	Met *	Cys	Val *	Ile *	Leu *	Phe *	Tyr **	Trp *	AAS *** (%)	Reference
*Zonocerus variegatus*	48.4	39.2	60.6	81.9	30.7	46.7	133.7	43.0	44.9	36.6	18.9	6.5	35.4	36.7	50.6	30.5	25.3	-	66.4	[43]
*Periplaneta americana* L.a	40.0	20.0	51.0	-	36.0	45.0	130	65.0	71.0	61.0	36.0	20.0	65.0	31.0	56.0	31.0	69.0	6.0	76.3	[44]
*Rhynchophoris phoenicis* (larvae)	45.0	38.9	79.2		30.6	39.0	156.0	50.1	47.2	52.5	19.7	20.2	35.0	39	54.2	47.5	29.0	-	71.9	[45]
*Sciphophorus acupunctatus* (larvae)	53.5	14.7	44.0	-	40.4	-	-	-	-	-	20.2	26.7	62.0	48.2	78.2	46.1	63.5	8.1	83.4	[46,47]
*Ephydra hians* (larvae)	55.0	10.0	-		49.0	-	-	-	-	-	19.0	-	61.0	40.0	74.0	54.0	51.0	7.1	74.3	[48]
*Hoplophorion monograma*	55.0	15.0	-		45.0	-	-	-	-	-	19.0	-	74.0	41.0	77.0	47.0	90.0	9.6	84.1	[48]
*Atta mexicanah*	49.0	25.0	-		43.0	-	-	-	-	-	34.0	-	64.0	53.0	80.0	88.0	47.0	6.0	89.3	[48]
*Liometopum apiculatumd*	58.0	29	50.0		42.0	-	-	-	-	-	18.0	14.0	60.0	49.0	76.0	39.0	68.0	8.0	88.0	[48]
*Macrotermes bellicosus*	54.2	51.4	69.4		27.5	-	-	-	-	-	7.5	18.7	73.3	51.1	78.3	43.8	30.2	14.3	97.3	[46]
*Bombyx mori* (larvae)	47.3	25.8	41.9	-	31.2	36.6	100.0	34.4	60.2	45.2	14.0	8.6	40.9	32.3	52.7	29.0	31.2	7.5	64.8	[49]
*Acheta domesticus* (adults)	53.7	23.4	61.0	-	36.1	49.8	104.9	56.1	50.7	87.8	14.6	8.3	52.2	45.9	100.0	31.7	48.8	6.3	78.2	[49]
*Boopedon flaviventrish*	55.0	24.0	-		44.0	-	-	-	-	-	18.0	-	57.0	47.0	88.0	41.0	74.0	6.0	83.1	
Fish (*Clarias anguillaris*)	50.2	11.8	47.8	70.4	20.8	19.2	118	24.5	31.1	24.8	23.4	7.3	28.0	25.8	64.7	38.7	24.6	-	-	[50]
Beef	45	20	33	52	25	27	90	28	24	30	16	5.9	20	16	42	24	22	-	-	[51]

* Denotes essential amino acids. ** Denotes semi-essential amino acids. *** Average Amino acid Score based on the egg’s amino acids as reference.

**Table 7:** The Bioactive peptides from various insects and their potential health benefits.

Insect Source	Enzyme Used	Identified Peptides	Bioactivity	Reference
Silkworm/domestic silk moth (*Bombyx mori*)	Pepsin, trypsin, R-chymotrypsin	simulated the human gastrointestinal hydrolysate	ACE inhibitory effects, with 100% activity in hydrolysates	[85]
Cotton leafworm (*Spodoptera littoralis*)	Pepsin, trypsin and α-chymotrypsin	Ala-Val-Phe	In vitro ACE inhibitory activity (IC50: 2123 μM)	[95]
Mealworm larva (*Tenebrio molitor*)	Alcalase	Tyr–Ala–Asn	ACE inhibitory activity (IC50: 0.017 mg/mL)	[89]
Silkworm pupae (*Bombyx mori*)	Alcalase, Prolyve, Flavourzyme, Brewers Clarex	SWFVTPF, NDVLFF	Antioxidant activity (ROS reduction, superoxide dismutase (SOD) expression and glutathione (GSH) production activity)	[90]
Tropical house crickets (*Gryllodes sigillatus*)	Alcalase-generated protein hydrolysates	YKPRP, PHGAP, VGPPQ	Anti-hypertensive, anti-glycemic, anti-inflammatory activities	[91]
Black soldier fly (*Hermetia Illucens*)	--------------------------	Hill-Cec1, Hill-Cec10	Antimicrobial activity against various pathogens	[96]
Silkworm pupae (*Bombyx mori*)	Acidic protease, Neutral protease	FKGPACA, SVLGTGC	Antioxidant activity, ABTS radical scavenging	[97]
Crickets (*Gryllus bimaculatus*)	Alcalase	TEAPLNPK, EVGA, KLL, TGNLPGAAHPLLL, AHLLT, LSPLYE, AGVL, VAAV, VAGL, QLL	Antioxidant activity	[98]
White-spotted flower chafer larva (*Protaetia brevitarsis*)	Flavourzyme	Ser-Tyr, Pro-Phe, Tyr-Pro-Tyr, Trp-Ile	ACE inhibitory activity, NO production in cells	[99]
Brown seaweed (*Laminaria digitate*)	Hydrolysates, fermentation generated peptides or generated from high-pressure processing	YIGNNPAKGGLF, IGNNPAKGGLF, and others (130 in total)	ACE-1 inhibitory activity, Potential DPP-IV inhibition	[100]

**Table 8:** Functional properties of edible insects.

Protein Source	WAC (%)	OAC (%)	FC (%)	FS (%)	EC (%)	ES (%)	Reference
Soy	300	345	68.66	nd	84.73	82.40	[104]
Mung bean	333	300	89.66	80.83	72.03	66.50	[105]
Lentil	447	154	235	nd	25	nd	[69]
Chickpea	131.6	109.3	46.3	39.2	48.8	45.1	[105]
Egg White	168	135	159.1	145.4	19.68 m^2^/g	60.01 m^2^/g	[101]
Whey Protein	-	-	-	23.7	4654 m^2^/g	23.2 h	[106]
Insects							
African migratory locust (*Locusta migratoria*)	nd	nd	459	55.76	55.19	nd	[101]
Bombay locust (*Patanga succincta*)	nd	nd	8.57	98.72	29.23 m^2^/g	15.67 m^2^/g	
Desert locust (*Schistocerca gregaria*)	218	322	32	6.17	67.78	50.41	[102]
Tropical house cricket (*Gryllodes sigillatus*)	344	333	99	92	72.62	38.3	[69]
Mealworm (*Tenebrio molitor*)	395	274	32.67	30.33	66.6	51.31	

All the data are expressed as a percentage unless stated otherwise. nd: not detectable. WAC: Water absorption capacity. OAC: Oil absorption capacity. FC: Foam capacity. FS: Foam stability. EC: Emulsifying capacity. ES: Emulsifying stability.
